# Comparison of invasive and non-invasive measurements of cardiac index and systemic vascular resistance in living-donor liver transplantation: a prospective, observational study

**DOI:** 10.1186/s12871-023-02302-x

**Published:** 2023-11-04

**Authors:** Hye-Yeon Cho, Ho-Jin Lee, In Eob Hwang, Hyung-Chul Lee, Won Ho Kim, Seong-Mi Yang

**Affiliations:** 1 Department of Anesthesiology and Pain Medicine, Seoul National University College of Medicine, Seoul, Republic of Korea; 2 Department of Anesthesiology and Pain Medicine, Nowon Eulji Medical Center, Eulji University, Seoul, Republic of Korea; 3 Department of Anesthesiology and Pain Medicine, Seoul National University Hospital, Seoul, Republic of Korea; 4 Department of Anesthesiology and Pain Medicine, Asan Medical Center, 88, Olympic-ro 43-gil, Songpa-gu, Seoul, 05505 Republic of Korea

**Keywords:** Blood pressure monitors, Cardiac output, Liver transplantation, Monitoring, Pulmonary artery catheterization, Pulse wave analysis, Vascular resistance

## Abstract

**Background:**

Based on the controversy surrounding pulmonary artery catheterization (PAC) in surgical patients, we investigated the interchangeability of cardiac index (CI) and systemic vascular resistance (SVR) measurements between ClearSight™ and PAC during living-donor liver transplantation (LDLT).

**Methods:**

This prospective study included consecutively selected LDLT patients. ClearSight™-based CI and SVR measurements were compared with those from PAC at seven LDLT-stage time points. ClearSight™-based systolic (SAP), mean (MAP), and diastolic (DAP) arterial pressures were also compared with those from femoral arterial catheterization (FAC). For the comparison and analysis of ClearSight™ and the reference method, Bland-Altman analysis was used to analyze accuracy while polar and four-quadrant plots were used to analyze the trending ability.

**Results:**

From 27 patients, 189 pairs of ClearSight™ and reference values were analyzed. The CI and SVR performance errors (PEs) exhibited poor accuracy between the two methods (51.52 and 51.73%, respectively) in the Bland-Altman analysis. CI and SVR also exhibited unacceptable trending abilities in both the polar and four-quadrant plot analyses. SAP, MAP, and DAP PEs between the two methods displayed favorable accuracy (24.28, 21.18, and 26.26%, respectively). SAP and MAP exhibited acceptable trending ability in the four-quadrant plot between the two methods, but not in the polar plot analyses.

**Conclusions:**

During LDLT, CI and SVR demonstrated poor interchangeability, while SAP and MAP exhibited acceptable interchangeability between ClearSight™ and FAC.

## Background

Hemodynamic instability frequently occurs during liver transplantation due to its surgical features, which include massive bleeding, manipulations of major vessels, and reperfusion of liver graft, as well as the recipient’s features, such as reduced systemic vascular resistance (SVR) and ventricular response [1]. Therefore, pulmonary artery catheterization (PAC) for continuous hemodynamic monitoring has traditionally been used in liver transplantation [2, 3]. However, PAC is an invasive procedure that potentially causes severe complications during its insertion or maintenance, such as pulmonary artery injury and ventricular arrhythmia [4]. Thus, its utility in surgical patients remains controversial, requiring a less-invasive hemodynamic monitoring method [5–8].

Several non-invasive hemodynamic monitoring techniques have been studied in various perioperative settings [9, 10]. In liver transplantation, several comparative studies have compared non-invasive cardiac output (CO) monitoring with thermodilution measurement via a PAC; however, these studies failed to yield satisfactory accuracy [11–15]. ClearSight™ (Edwards Lifesciences, Irvine, CA, USA) is a continuous, non-invasive finger-cuff arterial pressure monitoring device that uses photoplethysmographic technology [16] and enables the continuous measurement of CO through arterial pulse waveform analysis [9, 10, 16]. Previous studies have reported that finger arterial pulse analysis displays clinically acceptable interchangeability with conventional invasive hemodynamic monitoring in cardiac surgery and intensive care [17–19].

However, in liver transplantation, the interchangeability between ClearSight™ and conventional invasive hemodynamic monitoring has not yet been reported. Thus, we aimed to investigate their interchangeability by comparing hemodynamic variables, such as the cardiac index (CI), SVR, and blood pressure, obtained from ClearSight™ with those from conventional invasive hemodynamic monitoring via PAC and femoral arterial catheterization (FAC) during liver transplantation. We expect our results from liver transplantation to also provide valuable information regarding the usefulness of ClearSight™ in other major surgeries that are potentially complicated by hemodynamic instability.

## Methods

### Patients

This single-center, prospective, observational study was approved by our institutional review board (IRB No.2104-037-1209) and registered on the ClinicalTrials registry (NCT04909645; date of registration: 02/06/2021). This study was conducted in accordance with the principles of the 2013 Helsinki Declaration and followed good clinical practice guidelines. Patients who underwent scheduled living-donor liver transplantation (LDLT) were enrolled consecutively between May and December 2021. All patients received both verbal and written explanations of the trial and provided written informed consent. We excluded patients with persistent arrhythmias and a history of upper-extremity artery occlusion. Patients who had a perm catheter in the right internal jugular vein as well as contraindications for PAC [4], such as right-sided endocarditis, tumors, right-sided valve disease, and left bundle branch block, were also excluded.

### Anesthetic management

After routine vital monitoring, general anesthesia was induced using a bolus injection of propofol (1.0–2.0 mg/kg) and a target-controlled infusion of remifentanil. Tracheal intubation was performed following adequate muscle relaxation achieved through the administration of rocuronium (1.2 mg/kg). Subsequently, volume-controlled ventilation was initiated at a tidal volume ranging from 6 to 8 mL/kg without the use of positive end-expiratory pressure while maintaining a fraction of inspiratory oxygen between 0.4 and 0.6. Anesthesia was maintained using sevoflurane and remifentanil. After inserting a 31-mm 20-gauge catheter (SuperCath™ 5, Medikit Co., Ltd., Tokyo, Japan) into the right radial artery, continuous arterial pressure monitoring was initiated, and a ClearSight™ monitor (Edwards Lifesciences, Irvine, CA, USA) was attached to the ipsilateral middle finger to measure CO and blood pressure using an EV1000™ monitor (Edwards Lifesciences, Irvine, CA, USA). Additionally, femoral arterial pressure monitoring was initiated after inserting a 3-French 8-cm catheter (Arterial Leader Cath, Vygon, Ecouen, France) into the right femoral artery. A pulmonary artery (PA) catheter (Swan Ganz CCOmbo CCO/SvO2™; Edward Lifesciences LLC, Irvine, CA, USA) was inserted through the right internal jugular vein. The tip of the PA catheter was confirmed to be correctly positioned in lung zone 3 using the pulmonary artery waveform, with the PA wedge pressure lower than the PA diastolic pressure. In addition, chest radiography was taken after the anesthetic induction. The PA catheter was connected to a Vigilance™ hemodynamic monitor (Edwards Lifesciences) to measure central venous pressure (CVP), CO, and CI. The pressure transducers (ClearSight™, pulmonary artery, CVP, femoral artery, radial artery) were zero-referenced to the right heart level. The ClearSight™ underwent auto-repeated self-calibration, while the Vigilance™ system was calibrated through pulmonary arterial blood sampling after confirming the PA catheter’s placement. And a fast flush test was conducted at 10-minute intervals to assess the quality of the femoral and radial arterial waveforms. Based on the attending anesthesiologist’s judgment, vasopressors (ephedrine, epinephrine, phenylephrine, vasopressin, and norepinephrine) were administered to maintain a femoral mean arterial pressure (MAP) of 65 mmHg or higher during the entire intraoperative period. Red blood cells were transfused to maintain a hemoglobin level > 8 g/dL. Fresh frozen plasma, platelets, and cryoprecipitate were transfused based on the rotational thromboelastometry results.

### CI, SVR, and blood pressure reference values

**Fig. 1:**
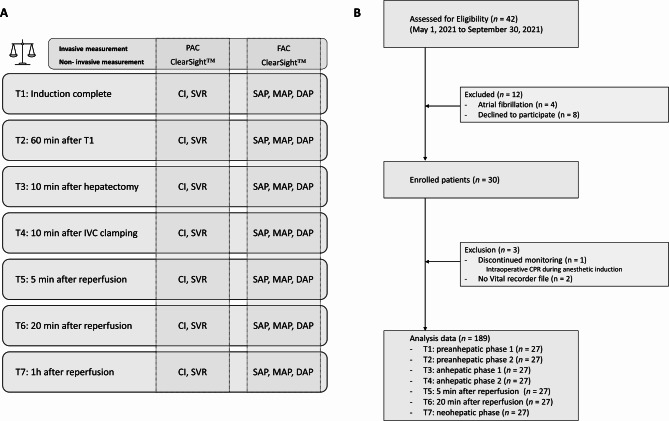
(**a**) The study protocol. (**b**) Flow diagram of the study. PAC; pulmonary artery catheterization, FAC; femoral artery catheterization; CI, cardiac index; SVR, systemic vascular resistance; SAP, systolic arterial pressure; MAP, mean arterial pressure; DAP, diastolic arterial pressure

CI measurement via the pulmonary artery thermodilution technique using a bolus injection of cold saline has not been implemented for over 20 years in our institution. Instead, continuous CI monitoring using a modified PAC with a heated filament has been used [20]. Therefore, we used the latter to obtain reference values for the CI and femoral arterial pressure (Fig. 1A), which is a more reliable blood pressure measure than radial arterial pressure during liver transplantation [3, 21]. The SVR reference value was calculated using the CVP value obtained from PAC and the MAP obtained from FAC (Fig. 1A).

## Data collection

Based on the liver transplantation stage [22], each hemodynamic variable obtained from the reference method and ClearSight™ was recorded at the following seven time points (Fig. 1A): T1 (preanhepatic 1), induction complete; T2 (preanhepatic 2), 60 min after T1; T3 (anhepatic 1), 10 min after completion of recipient hepatectomy; T4 (anhepatic 2), 10 min after inferior vena cava (IVC) clamping; T5 (reperfusion), 5 min after reperfusion; T6 (neo-hepatic 1): 20 min after reperfusion; and T7 (neo-hepatic 2): 1 h after reperfusion. Continuous infusion of vasopressor and core temperature, which potentially affect the accuracy of finger arterial pulse analysis, were also recorded at each time point [23].

### Statistical analysis

Based on previous studies on the accuracy of ClearSight™-derived CI in other surgeries [24, 25], we aimed to recruit a total of 30 patients, assuming a 10% dropout rate.

Bland–Altman analysis [26] was used to compare measured values between the reference method and ClearSight™, and the results are shown with bias and a 95% limit of agreement. The reference values for the CI and SVR were calculated using values obtained from PAC, and those for systolic arterial pressure (SAP), MAP, and diastolic arterial pressure (DAP) were those measured from FAC. Percentage error (PE) was calculated using Critchley and Critchley’s formula (1.96 × standard deviation/mean); for PEs < 30%, ClearSight™ was considered interchangeable with the reference method [27, 28]. The limits of agreement were calculated by reflecting the data measured several times from one patient [29].

Trending ability was analyzed using polar and four-quadrant plots [27, 28, 30]. In the four-quadrant plot, the horizontal axis was divided into four zones based on the change in value from the reference method, and the vertical axis reflected the change in value from ClearSight™. A correct quadrant was defined as a case wherein both the value obtained from the reference method and that from ClearSight™ were positive or negative (the upper-right and lower-left quadrants), and the exclusion zone was set at 0.3 L/min/m^2^ for the CI and 10% for SAP, MAP, and DAP [11]. The concordance rate was calculated as the proportion of the correct quadrant across all points, and concordance rates > 92% were considered clinically acceptable [27]. In the polar plot analysis, the angle from the line of identity (y = x) and magnitude of change by vector length reflected the agreement between the two methods [30]. The values are presented as the mean angle bias and radial limits of agreement. When the angular bias was less than ± 5 and radial limits of agreement less than ± 30°, the trending ability was considered acceptable [28, 30].

R (version 4.1.1 with R packages; R development Core Team, Vienna, Austria) using the *moonBook* package [31], and SAS (version 9.4; SAS Institute, Cary, US) software were used for all statistical analyses. A two-sided P value < 0.05 was considered to have statistical significance.

## Results

A total of 30 patients were enrolled in this study, and 189 measurements from 27 patients were included in the final analysis (Fig. 1B). Data from three patients were excluded from the analysis due to the following reasons: cancellation of surgery due to ST-segment elevation revealed after anesthetic induction, failed PAC due to repetitive arrhythmia, and the occurrence of intraoperative cardiac arrest during the preanhepatic phase. The baseline characteristics and perioperative variables are shown in Table 1. The percentage of patients with intraoperative norepinephrine infusion was 93.6% (26/27). None of the patients received epinephrine, vasopressin, or phenylephrine during the operation.

**Table 1:** Patient characteristics and perioperative variables

Variables	*n* = 27
**Baseline variables**	
Sex, M/F, n (%)	19/8 (70.4/29.6)
Age, years	59.2 ± 9.2
Height, cm	165.5 ± 8.6
Weight, kg	63.5 ± 10.5
Body-mass index, kg/m²	23.1 ± 2.8
MELD score	7.8 (6.8–10.2)
Child Pugh class A/B/C, n (%)	19 (70.4) / 6 (22.2) / 2 (7.4)
Etiology	
Viral-related liver cirrhosis, n (%)	19 (70.4)
Non-viral-related liver cirrhosis, n (%)	5 (18.5)
Other, n (%)	3 (11.1)
**Perioperative variables**	
Cold ischemic time, min	108.7 ± 39.3
Warm ischemic time, min	36.3 ± 15.8
Partial/Total IVC clamping, n	13 (48.1) / 14 (51.9)
IVC clamping duration, min	29.0 (20.0–39.5)
Anesthesia time, min	470.0 (425.0–532.5)
Estimated blood loss, mL	2630.0 (1250.0–4900.0)
Crystalloid, mL	4850.0 (3950.0–5675.0)
20% albumin, mL	350.0 ± 237.4
Patients with RBC transfusion, n (%)	19 (70.4)
Patients with FFP transfusion, n (%)	8/ (29.6)
Patients with plateletpheresis transfusion, n (%)	3 (11.1)

Values are presented as the mean ± SD, median (Q1, Q3), or number (%)

MELD: model for end stage liver disease, IVC: inferior vena cava, RBC: red blood cell, FFP: fresh frozen plasma

**Fig. 2:**
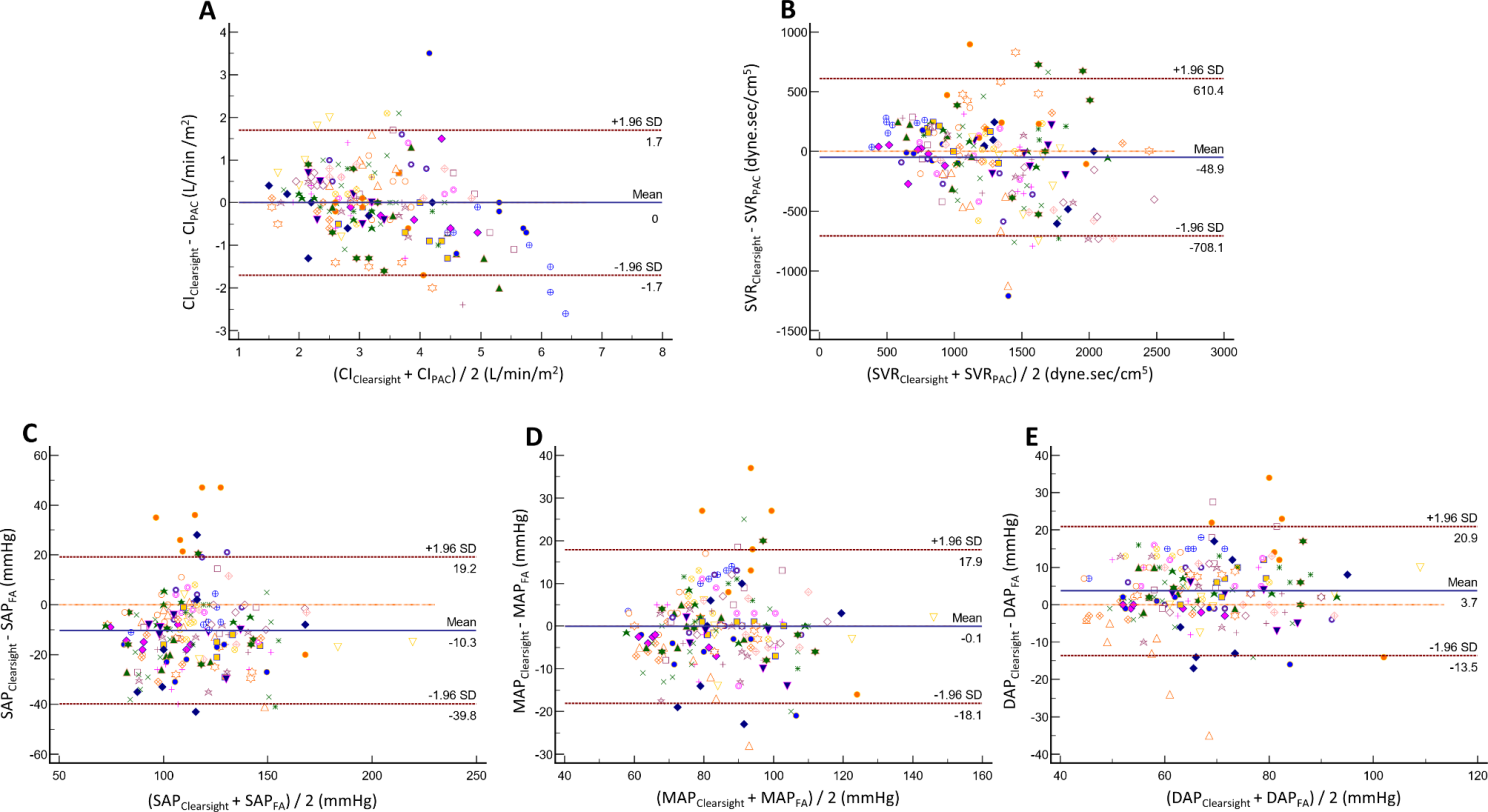
(**a**) Bland–Altman analysis comparing the CI measured using ClearSight™ with that using PAC, showing multiple measurements per subject. (**b**) Bland–Altman analysis comparing SVR measured using ClearSight™ with that using PAC, showing multiple measurements per subject. (**c**) Bland–Altman analysis comparing SAP measured using ClearSight™ with that using FAC, showing multiple measurements per subject. (**d**) Bland–Altman analysis comparing MAP measured using ClearSight™ with that using FAC, showing multiple measurements per subject. (**e**) Bland–Altman analysis comparing DAP measured using ClearSight™ with that using FAC, showing multiple measurements per subject. The blue line indicates the mean bias, and the dashed lines indicate the 95% limits of agreement in each analysis. CI, cardiac index; PAC, pulmonary artery catheterization; SVR, systemic vascular resistance; SAP, systolic arterial pressure; MAP, mean arterial pressure; DAP, diastolic arterial pressure, FAC; femoral artery catheterization; SD, standard deviation

The Bland–Altman analyses are shown in Fig. 2. The bias and 95% limits of agreement between the CIs from PAC and those from ClearSight™ were 0 L/min/ m^2^ and − 1.70 to 1.70, and those between the SVRs from PAC and those from ClearSight™ were − 48.87 dyne.s/cm^5^ and − 708.08 to 610.35, respectively. The PEs of the CI and SVR exhibited poor accuracy (51.52% and 51.73%, respectively) (Table 2). The PEs of ClearSight™ for blood pressure measured at the femoral artery were 24.28%, 21.18%, and 26.26% for SAP, MAP, and DAP, respectively (Table 3; Fig. 2), indicating favorable accuracy.

**Table 2:** Hemodynamic data during the different stages of liver transplantation

	Overall	T1	T2	T3	T4	T5	T6	T7
	(N = 189)	(N = 27)	(N = 27)	(N = 27)	(N = 27)	(N = 27)	(N = 27)	(N = 27)
BT (°C)	35.5 ± 0.6	35.7 ± 0.5	35.8 ± 0.5	35.7 ± 0.5	35.4 ± 0.5	35.2 ± 0.5	35.3 ± 0.5	35.7 ± 0.6
Norepinephrine infusion, n (%)	126 (66.7)	7 (25.9)	11 (40.7)	15 (55.6)	23 (85.2)	23 (85.2)	23 (85.2)	24 (88.9)
CVP (mmHg)	5.4 (3.8–8.2)	6.1 (4.7–10.1)	5.1 (3.8– 6.3)	4.3 (2.7– 6.5)	3.8 (1.8– 6.0)	7.3 (5.5–10.4)	5.3 (4.0– 8.9)	7.8 (4.2– 8.2)
HR (bpm)	83.0 (69.5–98.0)	73.0 (61.5–82.0)	77.0 (65.5–91.0)	85.0 (68.8–98.5)	85.0 (69.0–104.0)	84.5 (77.0–100.0)	86.0 (78.5–97.5)	85.0 (74.2–102.5)
CI_PAC_ (L/min/ m^2^)	3.0 (2.4–3.9)	3.0 (2.7– 3.3)	2.7 (2.3– 3.6)	2.6 (2.0– 3.4)	2.4 (1.7– 3.0)	3.1 (2.4– 3.7)	4.1 (3.3– 4.8)	3.7 (2.9– 4.6)
CI_CS_ (L/min/ m^2^)	3.1 (2.6–4.0)	2.8 (2.5– 3.1)	2.9 (2.3– 3.2)	3.1 (2.5– 3.5)	2.6 (2.2– 3.0)	4.0 (3.2– 4.5)	3.5 (3.1– 4.2)	3.4 (2.8– 4.4)
SVR_PAC_ (dyne.s/cm^5^)	1181.5 (881.6–1616.1)	1161.3 (1026.0–1613.0)	1345.5 (997.2–1680.7)	1384.3 (1061.4–1723.0)	1529.8 (1164.8–1935.0)	1296.6 (1095.3–1723.2)	829.2 (707.3–1056.4)	885.6 (685.6–1214.6)
SVR_CS_ (dyne.s/cm^5^)	1185.8 (846.5–1454.4)	1246.0 (935.4–1540.0)	1333.7 (1004.7–1708.4)	1234.8 (971.0–1466.8)	1395.6 (1029.0–1693.5)	1154.3 (830.2–1353.2)	927.1 (782.2–1205.7)	883.0 (801.1–1221.1)

Values are presented as the mean ± SD, median (Q1, Q3), or number (%). BT: body temperature, CVP: central venous pressure, HR: heart rate, CI_PAC_: cardiac index measured with pulmonary artery catheter, CI_CS_: cardiac index measured with ClearSight™, SVR_PAC_: systemic vascular resistance measured with pulmonary artery catheter, SVR_CS_: systemic vascular resistance measured with ClearSight™, T1: induction complete, T2: 60 min after T1, T3: 10 min after completion of recipient hepatectomy, T4: 10 min after inferior vena cava clamping, T5: 5 min after reperfusion, T6: 20 min after reperfusion, T7: 1 h after reperfusion

**Table 3:** Data on blood pressure during the different stages of liver transplantation

	Overall	T1	T2	T3	T4	T5	T6	T7
	(N = 189)	(N = 27)	(N = 27)	(N = 27)	(N = 27)	(N = 27)	(N = 27)	(N = 27)
SAP_FA_ (mmHg)	115.0 (104.0–135.0)	116.0 (106.0–144.5)	119.0 (105.2–137.0)	114.0 (100.5–124.0)	109.5 (96.5–120.5)	144.0 (121.0–155.5)	113.0 (102.0–125.2)	110.0 (104.5–118.2)
SAP_CS_ (mmHg)	109.0 (93.0–125.0)	109.0 (87.0–129.0)	114.0 (100.5–125.5)	108.0 (93.0–117.0)	104.0 (84.0–117.5)	131.0 (116.5–141.5)	107.0 (94.0–123.0)	104.0 (93.0–111.0)
MAP_FA_ (mmHg)	82.0 (72.0–94.0)	86.0 (73.5–102.5)	86.0 (76.2–94.5)	82.0 (75.5–87.0)	81.0 (70.5–86.5)	96.0 (84.0–104.0)	77.0 (71.0–89.2)	76.0 (70.0–79.8)
MAP_CS_ (mmHg)	83.0 (74.0–95.0)	85.0 (73.0–97.0)	89.0 (80.0–95.0)	83.0 (75.5–91.0)	80.0 (70.5–92.0)	96.0 (86.5–102.5)	80.0 (70.5–91.0)	79.0 (72.0–85.0)
DAP_FA_ (mmHg)	64.0 (56.0–75.0)	69.0 (58.0–83.0)	69.0 (61.8–79.0)	64.0 (61.5–74.0)	69.0 (58.2–76.0)	71.0 (61.5–76.0)	58.0 (52.0–66.8)	57.0 (52.0–62.0)
DAP_CS_ (mmHg)	69.0 ± 12.9	69.6 ± 13.8	72.5 ± 13.1	67.1 ± 10.4	67.3 ± 10.7	75.8 ± 15.9	66.2 ± 11.5	64.4 ± 11.5
PE_SBP_	24.28	16.56	24.12	24.51	25.99	24.39	24.43	30.04
PE_MBP_	21.18	19.54	19.15	19.78	19.85	20.26	20.86	28.02
PE_DBP_	26.26	18.58	20.49	23.43	22.72	24.49	23.74	42.11

Values are presented as the mean ± SD, median (Q1, Q3), or number (%). SAP_FA_: systolic arterial pressure measured at the femoral artery, SAP_CS_: systolic arterial pressure measured with ClearSight™, MAP_FA_: mean arterial pressure measured at the femoral artery, MAP_CS_: mean arterial pressure measured with ClearSight™, DAP_FA_: diastolic arterial pressure measured at the femoral artery, DAP_CS_: diastolic arterial pressure measured with ClearSight™, PE: percentage error. T1: induction complete, T2: 60 min after T1, T3: 10 min after completion of recipient hepatectomy, T4: 10 min after inferior vena cava clamping, T5: 5 min after reperfusion, T6: 20 min after reperfusion, T7: 1 h after reperfusion

**Fig. 3:**
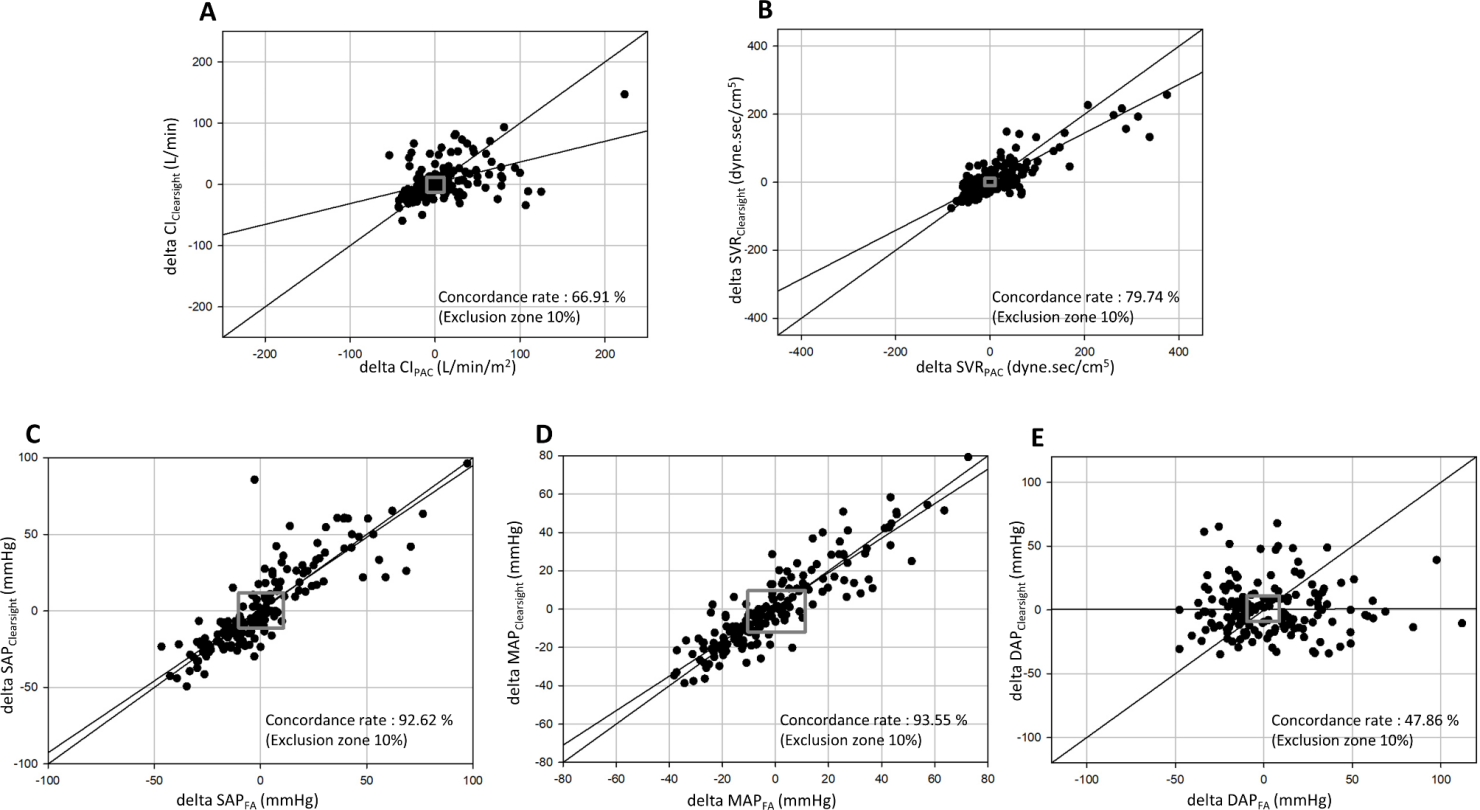
(**a**) Four-quadrant plot showing the concordance in the change in CI between ClearSight™ and PAC. (**b**) Four-quadrant plot showing the concordance in the change in SVR between ClearSight™ and PAC. (**c**) Four-quadrant plot showing the concordance in the change in SAP between ClearSight™ and FAC. (**d**) Four-quadrant plot showing the concordance in the change in MAP between ClearSight™ and FAC. (**e**) Four-quadrant plot showing the concordance in the change in DAP between ClearSight™ and FAC. The concordance rate was defined as the percentage of data points in which the change in the CI, SVR, SAP, MAP, and DAP of both methods goes in the same direction. The gray rectangle indicates the exclusion zone of 10%. CI, cardiac index; PAC, pulmonary artery catheterization; SVR, systemic vascular resistance; SAP, systolic arterial pressure; MAP, mean arterial pressure; DAP, diastolic arterial pressure; FAC; femoral artery catheterization

**Fig. 4:**
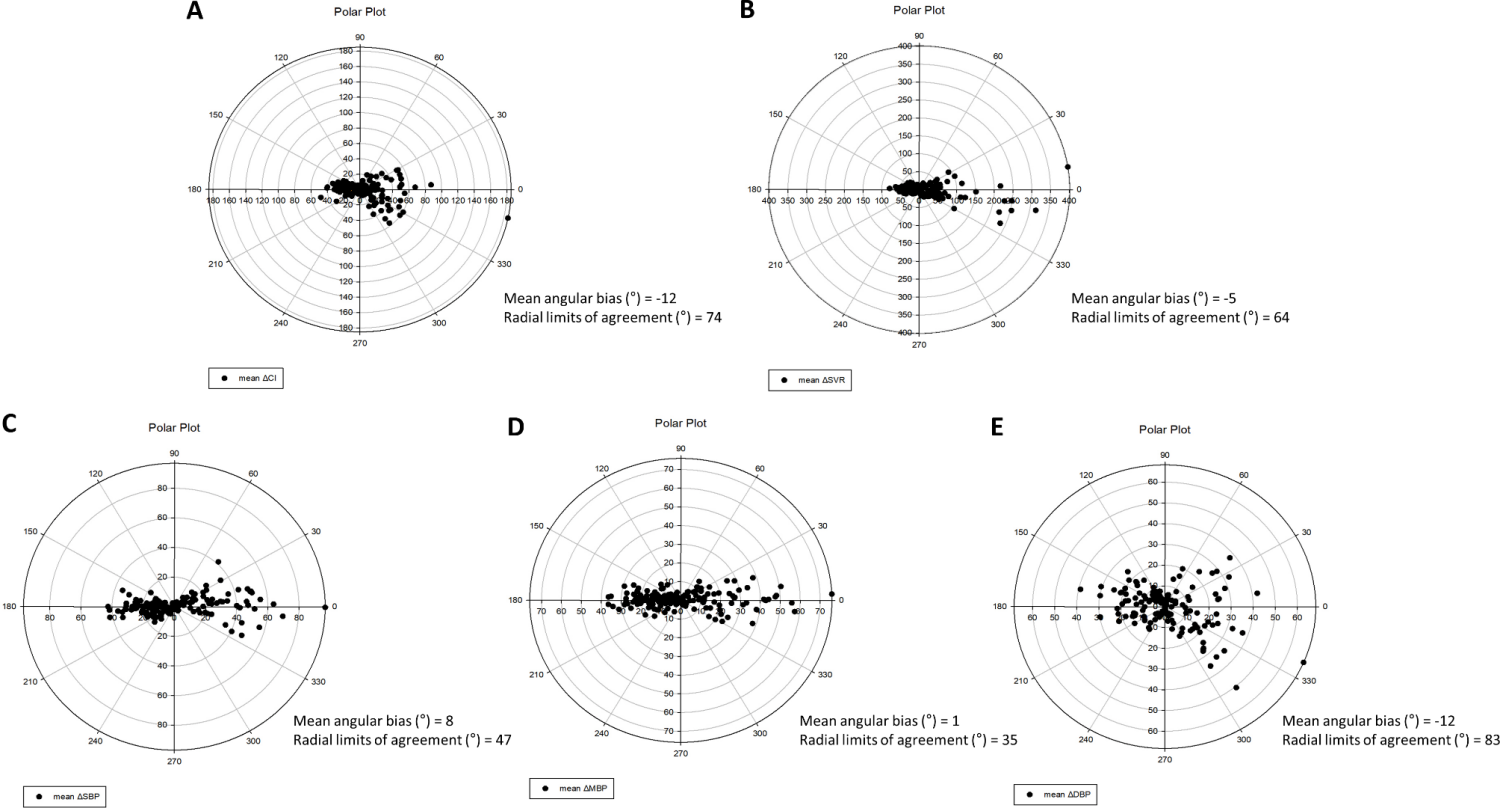
(**a**) Polar plots for examining the trending ability of CI change measured using ClearSight™ and PAC. (**b**) Polar plots for examining the trending ability of SVR change measured using ClearSight™ and PAC. (**c**) Polar plots for examining the trending ability of SAP change measured using ClearSight™ and FAC. (**d**) Polar plots for examining the trending ability of MAP change measured using ClearSight and FAC. (**e**) Polar plots for examining the trending ability of DAP change measured using ClearSight™ and FAC. CI, cardiac index; PAC, pulmonary artery catheterization; SVR, systemic vascular resistance; SAP, systolic arterial pressure; MAP, mean arterial pressure; DAP, diastolic arterial pressure; FAC; femoral artery catheterization

Four-quadrant plot and polar plot analyses were used to evaluate the trending ability of ClearSight™ for each reference method. In the four-quadrant plot analysis, the concordance rates of the CI and SVR measured between the two methods were 66.91% and 79.74%, respectively, exhibiting poor trending ability (Fig. 3). Similarly, the mean angular bias values (a radial limit of agreement) of the CI and SVR in the polar plot analysis were − 12° (74°) and − 5° (64°), respectively (Fig. 4). The concordance rates of SAP, MAP, and DAP between the two methods in the four-quadrant plot analysis were 92.62%, 93.55%, and 47.86%, respectively (Fig. 3). In the polar plot analysis, the mean angular bias values (a radial limit of agreement) of SAP, MAP, and DAP were 8° (47°), 1° (35°), and − 12° (83°), respectively (Fig. 4).

## Discussion

This study failed to demonstrate clinically acceptable interchangeability of CI and SVR between ClearSight™ and the continuous thermodilution method using a PA catheter with a heating filament during liver transplantation. SAP and MAP estimated using ClearSight™ displayed favorable accuracy compared with those measured using FAC and exhibited acceptable trending ability in the four-quadrant plot analysis. To the best of our knowledge, this study is the first to compare continuous hemodynamic monitoring between ClearSight™ and conventional invasive hemodynamic monitoring methods in liver transplantation.

PAC has been used as a therapeutic intervention for monitoring several hemodynamic parameters in high-risk surgical patients [32]. It has also been established as a routine practice in liver transplantation [33]. However, due to the development of less-invasive hemodynamic monitors and the risk of fatal complications associated with PAC [34], the usefulness of PAC in high-risk surgeries has recently been debated [34]. A recent retrospective study reported no difference in postoperative outcomes between hemodynamic management via PAC and arterial waveform analysis (Flotrac/Vigileo monitoring) in patients undergoing liver transplantation [35]. In a recent survey conducted by the Society for the Advancement of Transplant Anesthesia, approximately half of the anesthesiologists in high-volume centers indicated that < 50% of liver transplantation requires PAC [36]. Additionally, this survey reported a decreased routine use of PAC in liver transplantation compared with previous surveys [36]. On this premise, we attempted to investigate the interchangeability between ClearSight™ and PAC in this study.

Here, ClearSight™ did not exhibit clinically acceptable interchangeability with PAC in terms of CO and SVR. Similarly, previous studies have also reported poor interchangeability in the CI and SVR between PAC and other less-invasive arterial pulse wave analyses during liver transplantation [14, 15, 37]. Patients with cirrhosis undergoing liver transplantation possess unique characteristics, such as hyperdynamic circulation and low SVR, which potentially increase the inaccuracy of arterial pulse wave analysis [15, 38, 39]. Additionally, rapid changes in hemodynamic status during liver transplantation occur due to massive bleeding, manipulation of the IVC, inflammatory mediators after reperfusion of the graft, and the common use of vasoactive drugs that affect vascular compliance, thus negatively affecting the accuracy of arterial pulse wave analysis, including ClearSight™ [1, 3, 22]. Hypothermia, which is common during liver transplantation, also might have affected the accuracy of these methods [19, 39]. The point at which CO in ClearSight™ was calculated using the algorithm based on patient-related variables (age, sex, height, and weight) from the estimated blood pressure curve also might have affected the results [40]. Therefore, ClearSight™ would have failed to exhibit acceptable interchangeability in liver transplantation.

In addition to CO and SVR, we investigated the interchangeability of blood pressure measurements between ClearSight™ and FAC. Previous studies have predominantly investigated the interchangeability between ClearSight™ and radial arterial pressure [16, 24, 25, 37]. However, to our best knowledge, none have investigated the interchangeability between ClearSight™ and FAC-derived central arterial pressure in surgical patients. A recent prospective study using finger arterial pulse contour analysis reported a strong correlation between it and FAC-derived blood pressure in intensive care unit patients [41]. Our results demonstrate acceptable congruence of SAP, MAP, and DAP between ClearSight™ and FAC as well as an acceptable trending ability of SAP and MAP estimated using the four-quadrant plot. Although the polar plot analysis results did not satisfy predefined acceptable criteria, controversy regarding the superiority of the four-quadrant or polar plot analysis persists [42]. Since femoral arterial pressure is more reliable in blood pressure assessment than radial arterial pressure in critically ill patients [43], the favorable agreement and trending ability between ClearSight™ and femoral arterial pressure potentially indicate its usefulness in blood pressure monitoring in high-risk surgical patients. Additionally, since ClearSight™ displayed clinically acceptable interchangeability of MAP even in liver transplantation complicated by hemodynamic instability, our results potentially support the usefulness of ClearSight™ as a reliable pressure monitor in other high-risk surgeries.

This study has a few limitations. First, the time interval for CO measurement differed between ClearSight™ and PAC (20 s and 1 min, respectively). Therefore, each method might have presented values from different time points. In addition, since hemodynamic fluctuation in liver transplantation is common, the time delay between them might have negatively affected the consistency between the two methods [44]. Second, we could not conduct an a priori sample size calculation. An estimate of the study subjects’ CI was required to calculate the appropriate number of subjects in this study [45]; however, it was difficult to estimate due to severe hemodynamic instability in liver transplantation patients. Moreover, the distinctive characteristics of living donor liver transplantation posed challenges in recruiting a large number of patients, thereby necessitating the execution of this study on a small sample size. Nevertheless, despite the limitations in sample size, this study provides valuable insights into the feasibility of noninvasive methods for patients with hyperdynamic circulation during liver transplantation. Third, inotrope and vasopressor infusion at each time point was not considered. These drugs could have influenced the pulse contour analysis [5]. Fourth, our study predominantly focused on the interchangeability of CI, SVR, and AP estimations between ClearSight™ and conventional invasive hemodynamic monitors. Therefore, the advantages of conventional invasive hemodynamic monitoring, such as pulmonary arterial wedge pressure via PAC and arterial blood gas analysis via FAC, were not considered in this study. Finally, although the usefulness of transesophageal echocardiography (TEE) has been reported in liver transplantation [3], TEE results were not included in this study because TEE monitoring is used selectively in hemodynamic monitoring during liver transplantation in our institution.

In conclusion, ClearSight™ failed to demonstrate clinically acceptable interchangeability of CI and SVR estimations with the transpulmonary thermodilution method in LDLT. Therefore, continuous CI and SVR measurements using ClearSight™ cannot replace those using PAC in liver transplantation.

## Data Availability

The datasets generated during and analyzed during the current study are available from the corresponding author on reasonable request.

## Abbreviations

CI: Cardiac index
CVP: Central venous pressure
FAC: Femoral arterial catheterization
LDLT: Living-donor liver transplantation
MAP: Mean arterial pressure
PAC: Pulmonary artery catheterization
SVR: Systemic vascular resistance
